# Genetic Influence on Intergenerational Educational Attainment

**DOI:** 10.1177/0956797617707270

**Published:** 2017-07-17

**Authors:** Ziada Ayorech, Eva Krapohl, Robert Plomin, Sophie von Stumm

**Affiliations:** 1 MRC Social, Genetic and Developmental Psychiatry Centre, Institute of Psychiatry, Psychology & Neuroscience, King’s College London; 2 Department of Psychology, Goldsmiths, University of London

**Keywords:** intergenerational educational attainment, twin studies, behavioral genetics, polygenic score

## Abstract

Using twin (6,105 twin pairs) and genomic (5,825 unrelated individuals taken from the twin sample) analyses, we tested for genetic influences on the parent-offspring correspondence in educational attainment. Genetics accounted for nearly half of the variance in intergenerational educational attainment. A genomewide polygenic score (GPS) for years of education was also associated with intergenerational educational attainment: The highest and lowest GPS means were found for offspring in stably educated families (i.e., who had taken A Levels and had a university-educated parent; *M* = 0.43, *SD* = 0.97) and stably uneducated families (i.e., who had not taken A Levels and had no university-educated parent; *M* = −0.19, *SD* = 0.97). The average GPSs fell in between for children who were upwardly mobile (i.e., who had taken A Levels but had no university-educated parent; *M* = 0.05, *SD* = 0.96) and children who were downwardly mobile (i.e., who had not taken A Levels but had a university-educated parent; *M* = 0.28, *SD* = 1.03). Genetic influences on intergenerational educational attainment can be viewed as an index of equality of educational opportunity.

Educational attainment is key to a wide range of life outcomes, including employment, health, and even life expectancy (Bradley & Corwyn, 2002). Despite the profound benefits that accrue with educational qualifications, access to education—especially higher education—remains unequal (Boliver, 2013). Specifically, children whose parents obtained university degrees are more likely to register for higher education than are children from less educated families (Blanden & Machin, 2004).

Traditionally, differences in educational access have been attributed to sociopolitical constraints that result from social inequalities. Indeed, parents who have received more education have access to greater material and social resources that enable them to afford better opportunities for their children than less educated parents can afford (Bradley & Corwyn, 2002). For example, children from better educated families have more learning support in primary and secondary school, and they also receive tailored advice when preparing for university entry (Mackenbach et al., 2008; Pomerantz & Moorman, 2010).

A less frequently investigated factor that may contribute to parent-offspring similarity in educational attainment is genetics. Ubiquitous genetic influence is widely accepted for psychological traits (Knopik, Neiderheiser, DeFries, & Plomin, 2017), including academic achievement throughout the school years (Rimfeld, Ayorech, Dale, Kovas, & Plomin, 2016). However, the mechanisms contributing to intergenerational phenotypes are less understood.

One of the most widely studied parent-offspring associations is the link between family socioeconomic status (SES) and children’s educational outcomes (Sirin, 2005). Although this correlation is often interpreted as indicating that SES-related environmental factors directly cause the differences in children’s educational achievement, these differences are at least partly due to genetic influence (Krapohl & Plomin, 2016; Trzaskowski et al., 2014).

A game changer for genetic research in psychology is the genomewide polygenic score (GPS), which can be used to estimate genetic strengths and weaknesses of unrelated individuals from their DNA (Plomin & Simpson, 2013; Wray et al., 2014). A GPS aggregates the effects of thousands of DNA variants that were identified in corresponding genomewide-association (GWA) studies. A GPS from the first GWA study of years of education, which included more than 100,000 individuals (Rietveld et al., 2013), accounted for approximately 2% of the variance in years of education in independent samples, for 2.5% of the variance in family SES, and for almost 3% of the variance in children’s educational achievement (Krapohl & Plomin, 2016). This GPS based on the 2013 GWA report has demonstrated predictive potential for several socioeconomic outcomes, including mobility (Belsky et al., 2016; Conley et al., 2015; Domingue, Belsky, Conley, Harris, & Boardman, 2015), although effect sizes have been small.

Specifically, two recent studies using this GPS showed that adolescents’ GPS predicted educational attainment between and within families (Conley et al., 2015; Domingue et al., 2015). Furthermore, parents’ GPS was found to mediate the parent-offspring association in educational attainment, accounting for about 15% of this association (Conley et al., 2015). However contrary to previous findings regarding family SES (Turkheimer, Haley, Waldron, D’Onofrio, & Gottesman, 2003), the effects of adolescents’ GPS on educational attainment were not moderated by parents’ sociodemographic background (Conley et al., 2015).

In the research reported here, we extended these earlier studies in three ways. First, we used a concrete index of intergenerational educational attainment, comparing GPSs of offspring in four parent-offspring educational-attainment categories—downwardly mobile, upwardly mobile, stably educated, and stably uneducated (for definitions, see the Measures section). Second, we used a new GPS that explains twice as much variance in years of education as the GPS (Rietveld et al., 2013) used in the previous studies. This new GPS, derived from a 2016 GWA analysis of years of education in a sample of nearly 300,000 individuals, explains 3.9% of the variance in years of education in independent samples (Okbay et al., 2016). Finally, in addition to conducting DNA analyses, we used twin data to obtain the first well-powered estimate of the degree to which intergenerational educational attainment is heritable.

## Method

All participants were drawn from the Twins Early Development Study (TEDS), a longitudinal birth-cohort study of more than 15,000 twin pairs born in England and Wales between 1994 and 1996. Although there has been some attrition, more than 10,000 twin pairs remain actively involved in TEDS. The representativeness of the TEDS sample has been assessed at first contact in infancy, early childhood, middle childhood, and adolescence. At each of these ages, the distribution of ethnicity, family SES, and parental occupation in the TEDS sample has been shown to be representative of the United Kingdom (UK) population (Haworth, Davis, & Plomin, 2013; Kovas et al., 2007). For example, 92% of the families at first contact reported their ethnicity as White, which corresponds to the percentage of Whites (93%) in the UK population at that time. The TEDS families are also representative in terms of parents’ highest educational level (Bolton, 2012) and twins’ completion rate of A Levels (Department for Education, 2016), a key variable in our analyses. A Levels are a 2-year school option offered at the end of compulsory education in the United Kingdom at age 16, which is the first time students are free to choose whether or not to continue with formal education.

### Sample for the twin analyses

Data on the twins’ completion of A Levels, along with their parents’ highest educational qualification, were available for 12,210 individuals (6,105 twin pairs), after twins with a severe medical or psychiatric history or unknown zygosity were excluded. Zygosity was determined by parents’ responses on a questionnaire that is more than 95% accurate according to DNA testing (Price et al., 2000). When zygosity was unclear from this questionnaire, DNA testing was conducted. The sample for our analysis consisted of 2,128 monozygotic (MZ) twin pairs, 1,997 dizygotic (DZ) same-sex twin pairs, and 1,980 DZ opposite-sex twin pairs.

### Sample for the GPS analyses

Genomewide genotype data were obtained for a sample of unrelated individuals in TEDS (i.e., only one member of a given twin pair). The demographics of the genotyped subsample at first contact were representative of the United Kingdom. Individuals were removed from the GPS analyses if their ancestry was suspected to be non-European, as is standard practice (for more detail, see Selzam et al., 2016).

DNA was genotyped using Illumina HumanOmniExpressExome-8v1.1 arrays (Institute of Psychiatry, Psychology and Neuroscience Genomics & Biomarker Core Facility, London, United Kingdom) or Affymetrix GeneChip 6.0 DNA arrays (Affymetrix, Santa Clara, CA). The sample with genotype data consisted of 5,825 individuals (2,698 genotyped with Illumina and 3,127 genotyped with Affymetrix arrays). Genomewide genotypes from the two arrays were separately imputed using the Haplotype Reference Consortium (McCarthy et al., 2016) and the imputation software Minimac3 1.0.13 (Fuchsberger, Abecasis, & Hinds, 2015), which are available from the Michigan Imputation Server (https://imputationserver.sph.umich.edu). A series of quality checks were performed before merging data from the two arrays. Details about quality control and imputation have previously been described elsewhere for the same sample (Selzam et al., 2016).

After stringent pruning to remove markers in linkage disequilibrium (*r *^2^ > .1), and after we excluded eight genomic regions in high linkage disequilibrium so as to ensure that only genomewide effects were detected, we performed a principal components analysis to correct for possible stratification using a subset of 40,745 autosomal single-nucleotide polymorphisms (SNPs) that remained after we applied our quality-control criteria and that overlapped between the two genotyping arrays. We regressed the GPSs on the first 10 principal components and used the residuals in all subsequent analyses. More details of the quality-control and preprocessing procedures can be found in the Supplemental Material available online.

### Intergenerational educational attainment

Intergenerational educational attainment can be defined as the degree of similarity between the education levels of parents and their offspring (Kuntz & Lampert, 2013; Oberdabernig & Schneebaum, 2016). We operationalized intergenerational attainment by looking at parents’ attainment of an undergraduate degree and twins’ attainment of an A Level at age 18. Information on parental education was collected when participants in the TEDS sample were first contacted, between 1995 and 1998. Children with at least one parent who had obtained a university degree were considered to have been born into a university-educated family. A questionnaire concerning A-Level and other post-age-16 qualifications, along with current employment categories, was sent to all TEDS families at the end of the academic school year when the twins reached age 18. The questionnaire was completed either by the twins themselves or by their parents.

These data were used to divide the sample into four groups: downwardly mobile, stably educated, upwardly mobile, and stably uneducated. The downwardly mobile group consisted of twins who had not completed A Levels but had been raised in families with at least one university-educated parent. The upwardly mobile group consisted of twins who had completed A Levels but whose parents had not attended university. The stably educated and stably uneducated groups included those twins whose educational path following compulsory schooling was similar to their parents’; specifically, the former group consisted of twins who had completed A Levels and who had a parent with a university degree, and the latter group consisted of twins who had not completed A Levels and who did not have a parent with a university degree.

For several of our analyses, we used dichotomous indices of downward and upward mobility, which were computed as follows. The downward-mobility variable took values of either 0 or 1 and was applied to only those individuals from families with at least one university-educated parent. Individuals who were downwardly mobile received a score of 1 on this variable, whereas those who were stably educated received a score of 0. The upward-mobility variable could also take values of 0 and 1, and it was applied to only those individuals from families with no university-educated parent. In this case, individuals who had completed A Levels, and thus were upwardly mobile, received a score of 1, whereas those who were stably uneducated received a score of 0.

### Statistical analyses

#### Twin analyses

Univariate twin analyses assess the relative genetic and environmental contributions to variance in a trait by comparing intraclass correlations between MZ twins, who share all of their genes, and DZ twins, who on average share 50% of their segregating genes (Rijsdijk & Sham, 2002). The extent to which correlations for MZ twin pairs are greater than correlations for DZ twin pairs serves as an index of heritability—the proportion of phenotypic differences that can be attributed to genetic differences (Knopik et al., 2017). When the available phenotypic data are categorical, the univariate model can be extended to a liability model that estimates additive genetic (*A*), shared environmental (*C*), and unique environmental (*E*) etiologies, assuming that binary variables reflect an unobserved normal distribution (Neale & Cardon, 1992). We followed this approach in twin analyses using our dichotomous indices of downward and upward mobility.

In addition, liability correlations, known as tetrachoric correlations, were computed using the concordance rates for intergenerational educational attainment in MZ and DZ twins. Comparing the tetrachoric correlations across MZ and DZ pairs allowed us to estimate genetic influences on intergenerational attainment. Similar correlations for MZ and DZ pairs would indicate that intergenerational educational attainment is predominantly driven by nongenetic factors shared within a family. That is, if one twin in a family was downwardly mobile (i.e., a parent had a university degree but the twin had not taken A Levels), the likelihood that the co-twin was also downwardly mobile would be the same for MZ and DZ pairs if intergenerational educational attainment is predominantly influenced by nongenetic factors. By contrast, if MZ twins were more similar in their intergenerational educational attainment than DZ twins were, this would imply genetic influences.

Tetrachoric correlations and *ACE* components were estimated with maximum likelihood using OpenMx (Boker et al., 2011). The liability threshold model has been described elsewhere in detail (Rijisdijk & Sham, 2002).

#### GPS analyses

GPS analyses tested whether aggregates of SNPs associated with number of years of education significantly predicted intergenerational educational attainment in our sample. A GPS sums genotypic values (0, 1, or 2) for each SNP weighted by its association in the discovery GWA sample. A GPS based on summary statistics (β weights and *p* values) from the 2016 GWA study of educational attainment (Okbay et al., 2016) was created for each of the 5,825 unrelated individuals in the genotyped sample. We used all matched SNPs (i.e., *p*-value threshold of 1.0) to compute these GPSs, irrespective of the nominal significance of the association of the SNPs with educational attainment. The resulting GPSs were normally distributed and were standardized to have a mean of 0 and a standard deviation of 1.0.

Separate logistic regression models were fitted to test the association between this GPS and intergenerational educational attainment. We used the Nagelkerke (1991) *r *^2^ to estimate the amount of variance explained. Analyses of variance (ANOVAs) tested mean GPS differences among the educational-attainment groups. Also, analyses of covariance tested whether GPS differences among the educational-attainment groups remained significant after controlling for previous academic performance, specifically, the twins’ grades on the UK General Certificate of Secondary Education (GCSE) examinations, which are administered at the end of compulsory education at age 16.

## Results

Of the total sample of 6,105 twin families, 1,790 families included at least one parent who was university educated (29%). By comparison, 6,304 of the 12,210 twins (52%) had completed A Levels. Thus, overall, the pursuit of higher education increased in the younger generation.

Of the 3,580 twins in the 1,790 families with at least one university-educated parent, 989 (28%) had not completed A Levels (the downward-mobility group). By contrast, of the 8,630 twins in the 4,315 families with parents who were not university educated, 3,713 (43%) had completed A Levels (the upward-mobility group). Of the 3,580 twins raised by university-educated parents, 2,591 (72%) had continued to A Levels (the stably educated group), and of the 8,630 twins whose parents were not university educated, 4,917 (57%) had not pursued A Levels (the stably uneducated group).

Table 1 shows the concordance of MZ and DZ twins for A-Level attainment separately for families with and without at least one university-educated parent. These data were used to calculate twin tetrachoric correlations. However, a rough index of twin similarity is the probandwise twin concordance (McGue, 1992). The MZ and DZ concordances were .94 and .82, respectively, in families with at least one university-educated parent, and .93 and .79 in families without a university-educated parent. These values suggest some genetic influence on intergenerational educational attainment.

**Table 1.** MZ and DZ Twin Concordances for A-Level Attainment in Children of University-Educated and Non-University-Educated Parents

Twin type	Families with a university-educated parent			Families without a university-educated parent		
	*n* pairs	Concordant	Discordant	*n* pairs	Concordant	Discordant
MZ	602	537 (89%)	65 (11%)	1,526	1,314 (86%)	212 (14%)
DZ	1,188	822 (69%)	366 (31%)	2,789	1,830 (66%)	959 (34%)

Note: MZ = monozygotic; DZ = dizygotic.

### Twin tetrachoric correlations

Twin tetrachoric correlations were derived from the data in Table 1 and analyzed using the liability threshold model in order to estimate genetic and environmental influences on intergenerational educational attainment and their confidence intervals (CIs). These correlations were greater for MZ than for DZ twin pairs, both for children with a university-educated parent (MZ: *r* = .91; DZ: *r* = .68) and for children without a university-educated parent (MZ: *r* = .91; DZ: *r* = .65). These results indicate that there is genetic influence on downward and upward intergenerational educational attainment. Because the DZ correlations were greater than half the MZ correlations, shared environmental influences can also be assumed.

### Twin ACE analyses

**Fig. 1.**
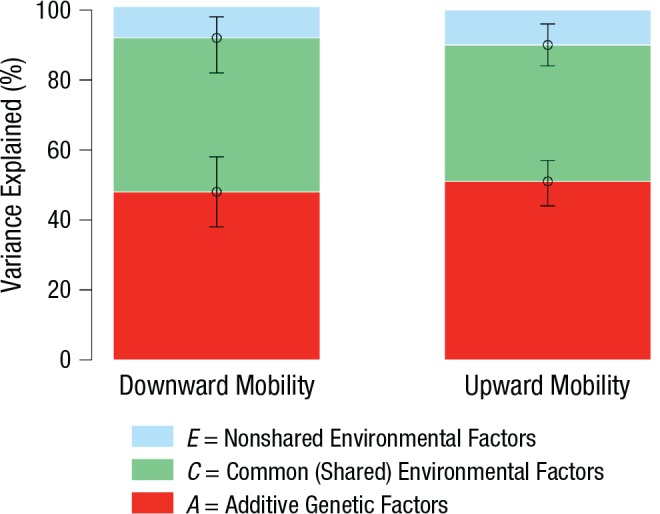
Results of the liability threshold models for downward mobility (left) and upward mobility (right); proportion of variance accounted for by additive genetic factors (*A*), shared environmental factors (*C*), and nonshared environmental factors (*E*). Downward mobility refers to the dichotomous variable for children from families with at least one university-educated parent; this model included the data from children who were either downwardly mobile (had not completed A Levels) or stably educated (had completed A Levels). Upward mobility refers to the dichotomous variable for children of parents without a university education; this model includes the data from children who were either upwardly mobile (had completed A Levels) or stably uneducated (had not completed A Levels). Error bars represent 95% confidence intervals.

Figure 1 shows the proportion of variance in intergenerational educational attainment that was estimated to be accounted for by genetic and environmental factors. The graphs indicate a substantial genetic influence, as approximately half of the phenotypic variance in liability was attributed to inherited DNA differences in both the upward-mobility analysis and the downward-mobility analysis. The influence of shared environmental factors (i.e., factors that contribute to similarities between twins growing up in the same home) was almost as large, accounting for approximately 40% of the variance. Nonshared environmental factors (i.e., factors that do not contribute to twin similarity) explained less than 11% of the variance in both analyses. (See Table S1 in the Supplemental Material for details.)

### GPS analyses

**Fig. 2.**
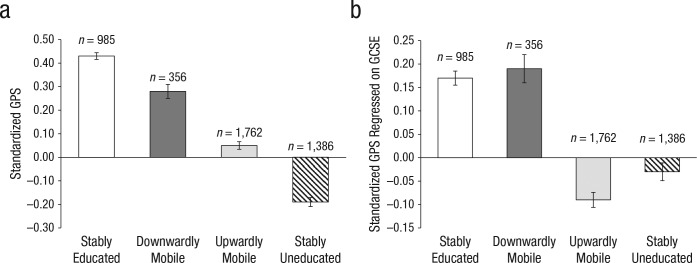
Mean standardized genomewide polygenic scores (GPSs) for the four educational-attainment groups: (a) uncorrected means and (b) means corrected for grades on the General Certificate of Secondary Education (GCSE). For each data bar, the sample size is shown. Error bars represent ±1 *SE*.

Figure 2a shows the mean GPSs for the four educational-attainment groups. Children who were downwardly mobile had a lower mean GPS (*M* = 0.28, *SD* = 1.03) than did children who were stably educated (*M* = 0.43, *SD* = 0.97), *F*(1, 1181*) =* 5.36, *p* < .001. Children who were upwardly mobile had a significantly higher mean GPS (*M* = 0.05, *SD* = 0.96) than did children who were stably uneducated (*M* = −0.19, *SD* = 0.97), *F*(1, 2759) = 43.6, *p* < .001. Children with a university-educated parent had a higher mean GPS (*M* = 0.39, *SD* = 0.98) than did children without a university-educated parent (*M* = 0.08, *SD* = 0.97) irrespective of whether they themselves went on to complete A Levels, *F*(1, 3242) = 48.8, *p* < .001. Similar results were found when analyses were rerun separately for mothers and fathers (see Figs. S1 and S2 in the Supplemental Material available online).

GPS accounted for a significant proportion of liability variance in both upward mobility (Nagelkerke *r *^2^ = .021, *p <* .001) and downward mobility (Nagelkerke *r *^2^ = .016, *p <* .001) in our independent sample of more than 5,000 unrelated individuals. A 1-*SD* increase in GPS was associated with a 36% increase in the odds of experiencing upward educational mobility in children who did not have a parent with a university degree (odds ratio, OR = 1.36, 95% CI = [1.25, 1.47]; *n* = 2,792). Similarly, a 1-*SD* increase in GPS was associated with a 29% decrease in the odds of experiencing downward educational mobility in children from families with at least one university-educated parent (OR = 0.71, 95% CI = [0.62, 0.82]; *n* = 1,200).

We also investigated the extent to which genetic influence on upward and downward educational mobility depended on previous school performance, assessed by GCSE grades (see Fig. 2b). After GPSs were adjusted for GCSE grades, the difference in mean GPS between the stably educated *(M* = 0.17, *SD* = 1.01) and downwardly mobile (*M* = 0.19, *SD* = 1.07) groups was no longer significant, *F*(1, 1047) = 0.035, *p* = .85. Likewise, the difference in mean GPS between the stably uneducated (*M* = −0.03, *SD* = 0.99) and upwardly mobile (*M* = −0.09, *SD* = 0.98) groups was no longer significant, *F*(1, 2194) = 2.659, *p* = .10. However, the difference in mean GPS between twins with a university-educated parent (*M* = 0.18, *SD* = 1.02) and twins without a university-educated parent (*M* = −0.06, *SD* = 0.98) remained significant, *F*(1, 3242) = 40.79, *p* < .001.

## Discussion

We have reported the first study on the genetics of intergenerational educational attainment that used both twin and genomic data. Our twin analyses indicate that half of the variance in intergenerational educational attainment can be attributed to genetic differences. These results demonstrate that the educational outcomes of parents and their offspring are similar for genetic as well as environmental reasons.

The results from our twin models were supported by our genomic analyses. Using a GPS derived from GWA results for adult educational attainment (Okbay et al., 2016), we observed significant mean GPS differences across four groups differing in intergenerational educational attainment. The highest and lowest mean GPSs were observed in the stably educated and stably uneducated groups, respectively—the GPSs were in between for offspring who were upwardly and downwardly mobile. This finding is in line with two prior studies on molecular genetic correlates of educational attainment that relied on a less powerful GPS: Conley et al. (2015) found evidence of genetic transmission in parent-child educational correlations, and Domingue et al. (2015) found that participants with higher polygenic scores were more likely to grow up in socially advantaged families.

By contrast to previous studies (e.g., Selzam et al., 2016), ours used Okbay et al.’s (2016) GPS to predict the attainment of educational qualifications rather than achievement in terms of school grades. School-leaving certificates, such as A Levels, regulate access to further education and, thus, affect career opportunities. Notwithstanding the importance of educational attainment for people’s life trajectories, our study is the first to test genetic influences, as measured by twin-based heritability and associations with the 2016 GPS, on an intergenerational phenotype of educational attainment.

Associations between GPSs and intergenerational attainment are likely to be mediated by many psychological characteristics, all of which are under substantial genetic influence. The most obvious candidate is prior academic achievement, which greatly informs children’s decision to go on to A Levels. In the current analyses, after we adjusted GPSs for the children’s academic performance at age 16 (GCSE grades), the difference in mean GPS between the stably educated and downwardly mobile groups was no longer significant, and neither was the difference in mean GPS between the stably uneducated and upwardly mobile groups. These results suggest that the effects of GPS on educational mobility are largely driven by children’s differences in prior academic performance. That said, the genetic effect of parents’ education level on children’s attainment remained. Future studies may explore other specific psychological mechanisms that explain the association between DNA and intergenerational educational attainment.

A noteworthy finding in the present study is that intergenerational educational attainment is influenced to a large extent by shared environmental factors. Although it is reasonable to assume that shared environment, such as the home that children grow up in or the schools that siblings attend, shapes educational trajectories, strong shared environmental influences, like those reported here, are rare in the psychological literature (Plomin, 2011). Our twin analyses estimated that shared environmental influences account for 40% of the variance in liability of intergenerational educational attainment, whereas estimates of shared environmental influences rarely exceed 20% for other education-related measures (Knopik et al., 2017).

The finding that there are genetic influences on intergenerational attainment suggests that some individuals who are born into socially disadvantaged families but surpass the constraints typically associated with low SES do this in part because of their genetic propensities. Indeed, we found higher mean GPS in the upwardly mobile group compared with the stably uneducated group, which indicates that children with more education-associated alleles went on to attain A-Level qualifications despite their familial environment.

A compelling implication of our results is that, to the extent that genetics is important, parent-offspring resemblance for educational attainment could be viewed as an index of environmental equality, rather than inequality, in society. This is because heritability estimates index the extent to which genetic differences account for phenotypic variance in a particular population with its particular mix of genetic and environmental influences. As environmental opportunities improve across a society, genetic influences are maximized, such that educational attainment is increasingly a function of individual characteristics and less a product of social conditions (Conley et al., 2015; Tucker-Drob, Briley, & Harden, 2013).

### Strengths and limitations

The present study benefited from a large sample size and inclusion of both twin and genomic analyses. Nonetheless, our results must be considered in light of three limitations, in addition to the general limitations of the twin method (Knopik et al., 2017).

First, our measure of educational attainment is a proxy measure given that the assessment of the twins’ A-Level attainment extended only through age 18 years, unlike our parental educational-attainment measure, which reflected adult attainment. Some twins who had not obtained an A-Level qualification at the time of the age-18 questionnaire may ultimately obtain a university degree, and others who had taken A Levels may fail to complete a university degree. That said, in Britain, fewer than 17% of students are admitted to university without A-Level qualification (personal communication, HESA, June 22, 2016), and only 6% of 2013–2014 university enrollees who obtained their A Levels failed to complete their degree (HESA, n.d.).

The second limitation is that educational attainment is partly conditioned by cohort changes in educational norms. Our analyses are based on a single European birth cohort, and the generalizability of our results outside of this population has yet to be formally tested.

Finally, although the GPS we used (Okbay et al., 2016) accounts for 3.9% of the variance in years of education, this GPS explains only about 6.5% of the heritability of years of education as estimated in twin studies (Selzam et al., 2016). This limited the potential effect size for our genetic analysis of intergenerational educational attainment. However, as the so-called missing-heritability gap closes, GPSs will explain more of the heritable variance in complex traits, and the predictive potential of GPSs in independent samples will improve.

### Conclusion

Our findings highlight the need for genetically sensitive studies of the factors that influence intergenerational educational outcomes and inequality. Leveraging genomic data to tackle questions about putatively social variables is key for understanding complex human behavior.

## Supplementary Material

Supplementary material
