# Supplementary material for: Genetic Influence on Intergenerational Educational Attainment
Source: Psychol Sci. 2017 Jul 17;28(9):1302–10. doi: 10.1177/0956797617707270 (PMC5595239; doi:10.1177/0956797617707270)
Supplement: Supplementary material [file Ayorech_Rev_Supplemental_Material.pdf]

Supplemental Material

Genetic influence on intergenerational educational attainment

Ziada Ayorech[1], Eva Krapohl[1], Robert Plomin[1], Sophie von Stumm[2*]

Additional methods:

Further details on genotyping

Figures:

Figure S1. Standardized GPS for each of the educational mobility groups when parental educational attainment is based on mother's university attainment only and GPS are conducted using all SNPs. *N*= sample size after exclusions (unrelated individuals); error bars = standard error. Stably educated= children of university educated parents who take A-levels; Downwardly mobile= children of university educated parents who do not take A-levels; Upwardly mobile= children of parents without a university degree who take A-levels; Stably uneducated= children of parents without a university degree who do not take A-levels.

Figure S2. Standardized GPS for each of the educational mobility groups when parental educational attainment is based on father's university attainment only and GPS are conducted using all SNPs. *N*= sample size after exclusions (unrelated individuals); error bars = standard error. Stably educated= children of university educated parents who take A-levels; Downwardly mobile= children of university educated parents who do not take A-levels; Upwardly mobile= children of parents without a university degree who take A-levels; Stably uneducated= children of parents without a university degree who do not take A-levels.

Genotyping

DNA for 4,649 individuals was extracted from saliva and buccal cheek swab samples and hybridized to Illumina HumanOmniExpressExome-8v1.2 genotyping arrays at the Institute of Psychiatry, Psychology and Neuroscience Genomics and Biomarker Core Facility. The raw image data from the arrays were normalized, pre-processed, and filtered in GenomeStudio according to Illumina Exome Chip SOP v1.4. (http://confluence.brc.iop.kcl.ac.uk:8090/display/PUB/Production+Version%3A+Illumina+Exome+Chip+SOP+v1.4). In addition, prior to genotype calling, 869 multi-mapping SNPs and 353 individuals with call rate <0.95 were removed. The ZCALL program was used to augment the genotype calling for samples and SNPs that passed the initial QC.

DNA from 3,665 individuals was extracted from buccal cheek swabs and genotyped on AffymetrixGeneChip 6.0 SNP genotyping arrays at Affymetrix, Santa Clara, California, USA using experimental protocols recommended by the manufacturer (http://www.affymetrix.com/support/downloads/manuals/genomewidesnp6_manual.pdf). The raw image data from the arrays were normalized and pre-processed at the Wellcome Trust Sanger Institute, Hinxton, UK for genotyping as part of the Wellcome Trust Case Control Consortium 2 (https://www.wtccc.org.uk/ccc2/) .Genotypes for the Affymetrix arrays were called using CHIAMO (https://mathgen.stats.ox.ac.uk/genetics_software/chiamo/chiamo.html).

After initial quality control and genotype calling, the same quality control was performed on the samples genotyped on the Illumina and Affymetrix arrays separately using PLINK (Chang et al., 2015), R (Purcell et al., 2007), and VCFtools (Danecek et al., 2011). Samples were removed from subsequent analyses on the basis of call rate (< 0.99), suspected non-European ancestry, heterozygosity, array signal intensity (> 4 SD from the mean) and relatedness. SNPs were excluded if the minor allele frequency was < 0.05%, if more than 1% of genotype data were missing, or if the Hardy Weinberg p-value was lower than 10^5. Non-autosomal markers and indels were removed. Association between the SNP and the array, batch, or plate on which samples were genotyped was calculated; SNPs with an effect p-value less than 10^3 were exclud

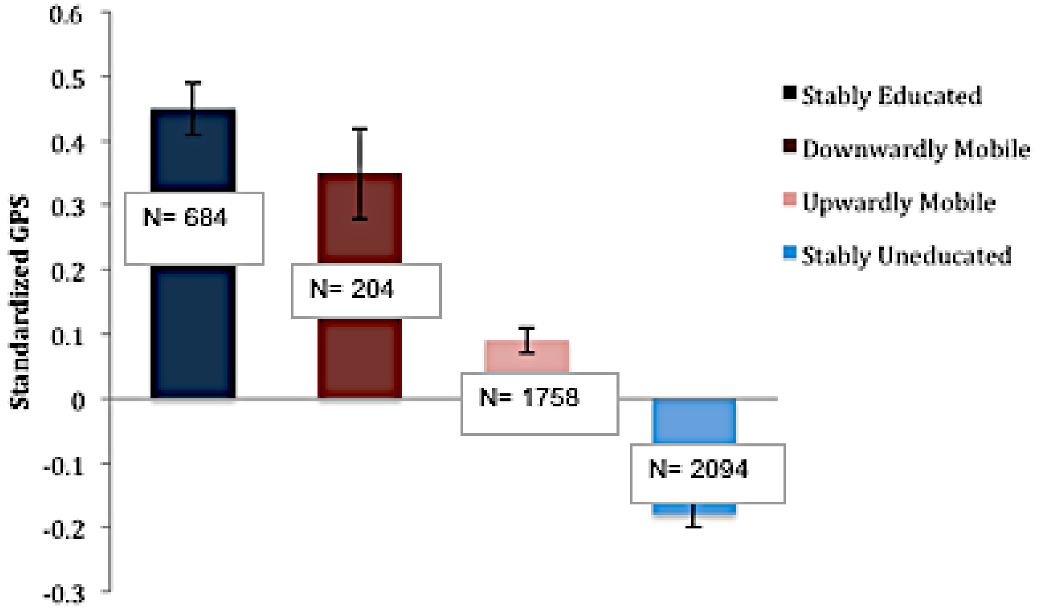

Figure S1. Standardized GPS for each of the educational mobility groups when parental educational attainment is based on mother's university attainment only and GPS are conducted using all SNPs (i.e., p-value threshold of 1.0).

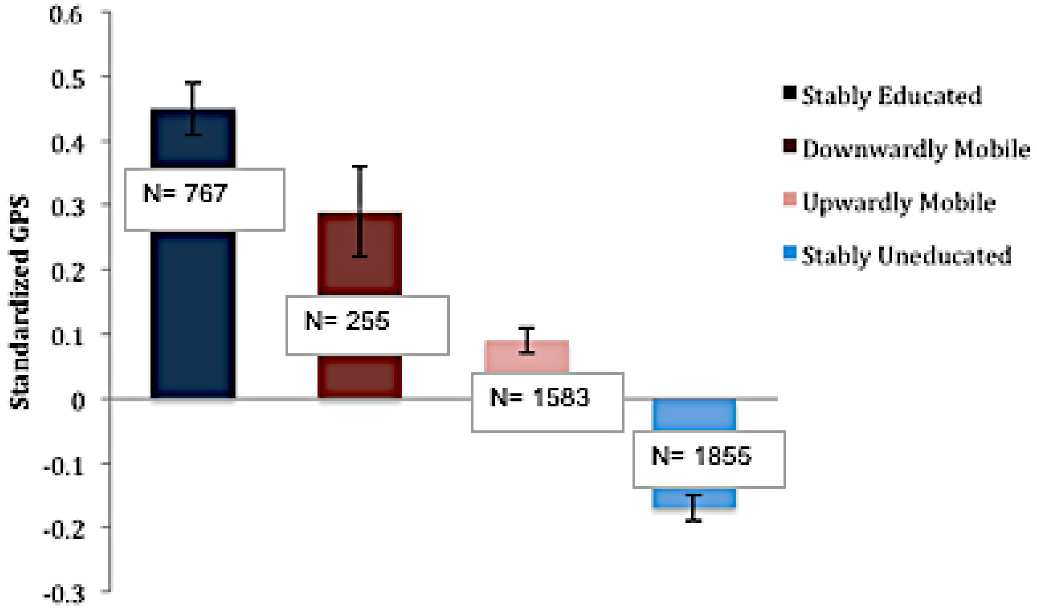

Figure S2. Standardized GPS for each of the educational mobility groups when parental educational attainment is based on father's university attainment only and GPS are conducted using all SNPs (i.e., p-value threshold of 1.0).

Table:

Table S1. Twin *ACE* estimates detailing the proportion of variance in intergenerational education attainment estimated to be accounted for by genetic and environmental factors. Downward mobility refers to the dichotomous variable for children from families with at least one university-educated parent, upward mobility refers to the dichotomous variable for children of parents without a university education. A= Additive genetic influence, C= Common (shared) environmental influence, E= Nonshared environmental influence.

Table S1. Twin *ACE* estimates detailing the proportion of variance in intergenerational education attainment estimated to be accounted for by genetic and environmental factors

				Twin tetrachoric correlations	
	**A**	**C**	**E**	**MZ**	**DZ**
**Downward Mobility**	0.48<br>(0.38-0.58)	0.44<br>(0.34-0.52)	0.09<br>(0.06-0.12)	0.91	0.68

				Twin tetrachoric correlations	
	**A**	**C**	**E**	**MZ**	**DZ**
**Upward Mobility**	0.51<br>(0.44-0.57)	0.39<br>(0.33-0.45)	0.10<br>(0.08-0.12)	0.91	0.65
